# Plate on Plate Osteosynthesis for the Treatment of Nonhealed Periplate Fractures

**DOI:** 10.1155/2014/367490

**Published:** 2014-03-11

**Authors:** Georgios Arealis, Vassilios S. Nikolaou, Andrew Lacon, Neil Ashwood, Mark Hamlet

**Affiliations:** ^1^Orthopedic Department, Queen's Hospital, Belvedere Road, Burton upon Trent, Staffordshire DE13 0RB, UK; ^2^2nd Department of Orthopaedics, School of Medicine, Athens University, Greece

## Abstract

*Purpose.* The purpose of this paper is to present our technique for the treatment of periplate fractures. *Methods.* From 2009 to 2012 we treated three patients. In all cases the existing plate was left and the new one placed over the existing. Locking screws were placed through both plates. The other screws in the new plate were used as best suited the fracture. *Results.* In all cases less than 6 months had passed between fractures. None of the original fractures had healed. Mean followup was 2 years. All fractures proceeded to union within 7 months. No complications were recorded. All the patients returned to their normal activities and were satisfied with the results of their treatment. *Conclusion.* Our plate on plate technique is effective for the treatment of periplate fractures. A solid fusion can be achieved at the new fracture site without disturbing the previous fixation.

## 1. Introduction 

Fractures are becoming more common with the increase in the ageing population. This has led to more patients requiring internal fixation to enable adequate healing and rehabilitation. Osteoporotic bone has a decreased healing capacity and a higher rate of implant failure [1, 2]. Additionally the interface between end of the plate and the osteoporotic bone is often a stress riser and may lead to periplate fracture [3]. This combined with an increased tendency to recurrent falls can all lead to an increasing frequency of peri-implant fractures.

The treatment of periprosthetic and perinail fractures is well reported in the literature [4, 5]. In contrast there is paucity of reports in the literature regarding the treatment of fractures around plates, especially when the first fracture has not healed. The purpose of this paper is to present our experience using a locking plate on top of the existing plate for the treatment of such fractures.

## 2. Patients and Methods

From 2009 to 2012 three consecutive female patients presented to our unit after sustaining a fracture around a locking plate (Table 1). In all cases the initial osteosynthesis was performed by another surgeon and less than six months had passed between the first osteosynthesis and the subsequent fracture around the plate. All procedures were performed by the senior author. All patients were followed up until fracture union. All plates, existing and second, were made of stainless steel and manufactured by Depuy-SYNTHES (Leeds, UK).

In all cases we made preoperative plans to leave the existing plate in situ and to utilize part of the original approach and extend it to allow a new plate to be placed partially over the existing one. The length and placement of the incision were similar to the one that would be used if only the second fracture existed. All operations were performed by the senior author.

After exposing the new fracture and finding the preexisting plate the new plate was laid to overlap it, thus allowing one to be able to see which holes in both plates accurately aligned. These screws were then removed from the original plate. New locking screws were placed through both plates and locked to the new “external” plate. An effort was made to use the original screw track in order not to weaken the bone with new drilling. Once the overlying plate section was fixed a standard screw was used to apply compression across the fracture site. When the plate is standing away from the bone it may be necessary to use a washer as spacer (case 2, Figure 2) or to contour the second plate 2 (case 3, Figure 3). The other screws in the new plate were used as best suited the fracture pattern as per standard procedure. In one case the last screw was unicortical to avoid creating a stress riser at the end of the second plate (case 2, Figure 2).

**Figure 1:**
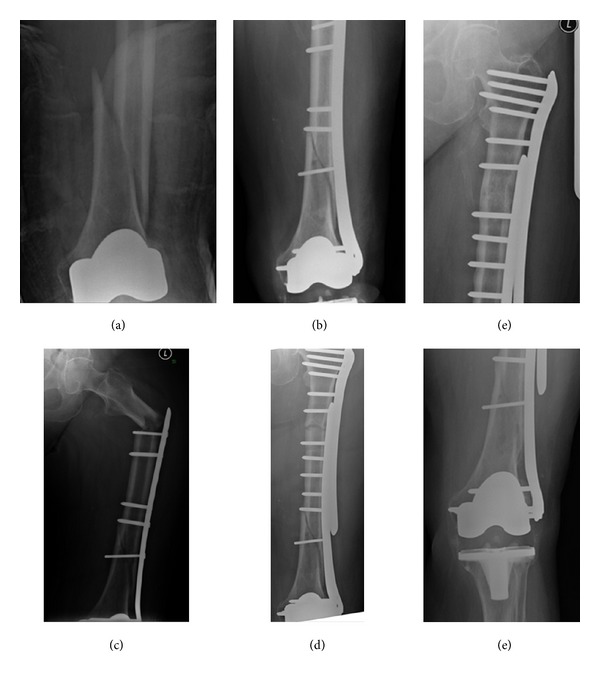
Case 1: (a) Periprosthetic fracture femur, (b) distal femoral plate (DFP) for the treatment of 1st fracture, (c) fracture proximal to the DFP plate, (d) postoperative X-ray, (e) healing of both fractures-DFP over DFP.

**Figure 2:**
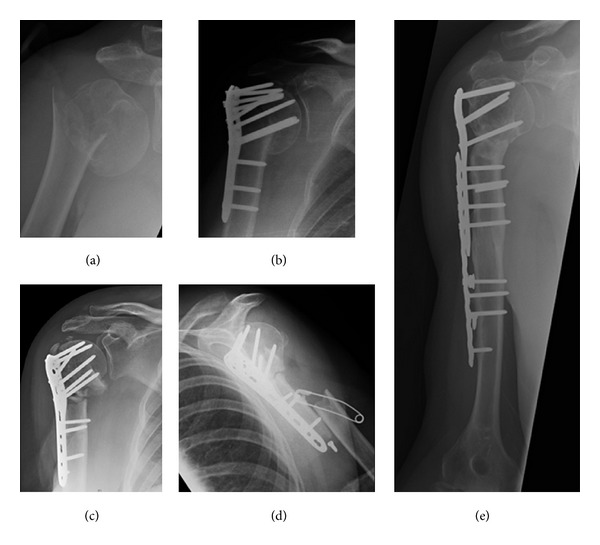
Case 2: (a) 1st fracture, (b) PHILOS plate, (c) correction osteotomy and replating with PHILOS plate, (d) periplate fracture 7 months after osteotomy, (e) LCP over PHILOS-healing of both fractures.

**Figure 3:**
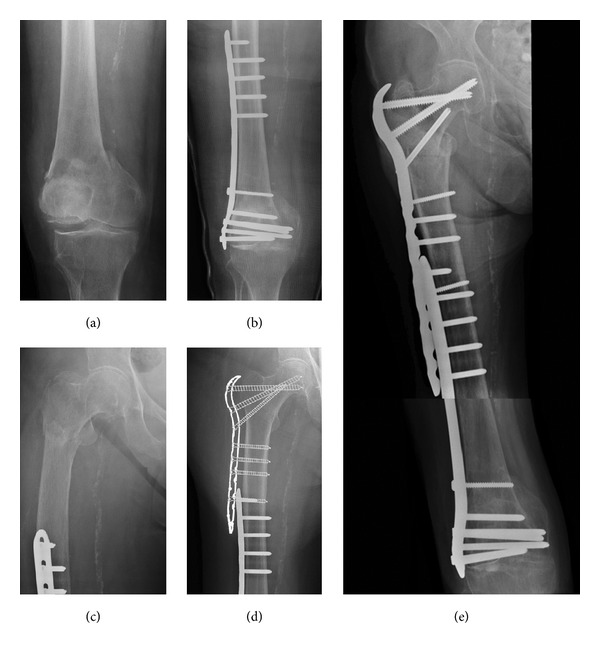
Case 3: (a) 1st fracture, (b) distal femoral plate (DFP) for supracondylar femoral fracture, (c) 2nd fracture-pertrochanteric, (d) using the prefracture X-ray for templating, (e) proximal femoral plate over DFP-healing of both fractures.

If this technique is used with a Depuy-SYNTHES (Leeds, UK) distal femoral plate then it should be noted that the plate is curved to match the contour of the femur. The ideal plate to sit on top is another distal femoral plate for the opposite femur as when placed upside down this will match the curvature and screw configuration (case 1, Figure 1).

## 3. Results

The mean follow-up time was 2 years (range: 18 months to 2.5 years). The age of our patients was 56, 85, and 93 years and all were female. Of them, one had a fracture distal to PHILOS plate (Depuy-SYNTHES, Leeds, UK) and 2 proximal to a distal femoral plate (Depuy-SYNTHES, Leeds, UK).

In all cases less than 6 months had passed between the first and the second fracture and none of the original fractures had healed. All fractures proceeded to union within 7 months from the second osteosynthesis.

No complications were recorded. We did not have any malunions. Also no superficial or deep wound infections were noted. All the patients returned to their normal activities and were satisfied with the results of their treatment.

## 4. Discussion

Fractures around existing implants stabilizing fractures are not very common and there are few reports in the current literature regarding their treatment [3]. Periprosthetic fractures following implants for hip, knee, shoulder, and elbow replacement are more common and usually effect the elderly population [4, 5]. The incidence of all types of peri-implant fractures will increase in the future because of the increase in the elderly population [1, 3].

All fractures in the elderly pose significant difficulties resulting from poor bone quality due to osteoporosis and reduced healing capacity. Both of these problems are evident in the increased failure rate of any implant applied to osteoporotic bone [6] even though delayed fracture healing is not always obvious [1]. Using conventional plates that rely on the friction between bone and plate for stability, for the treatment of osteoporotic fractures, can lead to failure due to screw pull-out. Locking constructs can overcome this disadvantage because stability is achieved through the locking interface between pate and screws and therefore it has been suggested that they are ideal for the treatment of osteoporotic fractures [7–11]. Cadaveric studies have also demonstrated that locking plates have an improved mechanical performance in torsional cycling loading over nonlocking constructs, especially in osteoporotic bone [12].

A major concern however, with the use of locking plates, is the rigidity of the locked screw-plate construct. The main fear is that the increased rigidity may potentially lead to delayed union or nonunion. This is more the case in diaphyseal fractures when the reduction is inadequate or when percutaneous techniques are used [8, 13]. For the same reason a risk of refracture exists after plate removal. This has been reported to be up to 4% if the plate is removed before 18 months [13]. It is not clear to what extent the danger of refracture is effected by the choice of compression or bridging plating and the number of locked screws and cortices used [14, 15].

There is a paucity of reports in the literature regarding periplate fractures that occur early, less than 18 months from the plating for the first fracture. This type of injury poses a great dilemma for the surgeon, especially if it happens in an elderly patient, which is usually the case. Removal of the first plate may lead to refracture at the first fracture site and requires an extensive approach. This is the case if both fractures are to be plated with one single longer implant. Another approach would be to place a second plate adjacent to the existing one, if there is enough space for the new plate to be secured. Unfortunately this will create a stress riser at the plate-plate region and may lead to fracture at this area. It has been shown that if two implants are used they must overlap in order to reduce the increased stress riser effect at their intersection [16]. Another way would be to place the new plate anterior or posterior to the existing but this may also cause problems. Firstly the screws of both plates may intersect causing difficulty in securing the second plate and secondly since the first plate is usually placed at the proper site it may be difficult to access the area anterior or posterior to the same region.

Plate material is not a restricting factor since recent reports have shown that stainless steel and titanium hardware can be mixed without any significant dangers [17]. The main fear has been that different materials could cause galvanic coupling corrosion. Despite the literature reports we used hardware manufactured from the same material in order to be more safe and in accordance with traditional AO principles [18, 19]. The other reason for choosing similar implants is their contour. As mentioned in the methods section similarly contoured plates can more easily be placed one on top of the other. Another potentially important issue is the material that the second plate is made of.

Another issue regarding the plate material is metallosis. Only two reports of metallosis following plating exist in the literature [20, 21]. Whether the cause is motion that leads to debris production from plate-screw interface and this results into metallosis and lysis and subsequent nonunion or the opposite is not clear, but it is likely that either mechanism can occur [21, 22]. In order to avoid plate on plate movement that could cause debris and local reaction it is very important to use locking screws at the plate on plate area and to reduce the fracture as anatomically as possible. Unfortunately metallosis can result very late, up to 7 years, following fracture fixation and long term follow-up is indicated.

The use of two plates in the treatment of fresh forearm fractures and for refractures when the length of the available plate was not adequate was described in the initial AO publications (1970) small fractures techniques book [23]. We perform a similar technique using locking plates. To the best of our knowledge our technique has never been described before and its use allows the surgeon to overcome all the aforementioned difficulties in the treatment of periplate fractures, especially in the elderly.

The disadvantages of our study are that it is retrospective, it is based on a series of heterogeneous fractures, and it involves only three patients making conclusions difficult to be drawn. Unfortunately, these fractures are not very common and a prospective study is very difficult to organize.

## 5. Conclusion

Our plate on plate technique is effective for the treatment of nonhealed periplate fractures, especially in elderly osteoporotic patients. Using this technique a solid fusion can be achieved at the new fracture without the need to disturb the previous fracture fixation. This method also decreases the morbidity of the periplate fracture surgery since the approach for its treatment is limited to the new fracture area without the need to access the preexisting plate-fracture area.

Further investigation, perhaps in the form of a multicentre study, is needed in order to fully evaluate this method and its results.

## Figures and Tables

**Table 1:** Case series and data.

Case	Age	Sex	Side	1st fracture site	1st fracture date	2nd fracture	Months to second fracture	Months to final healing	Types of plates
1	93	Female	Left	Midshaft peri THR	05/8/2009	Midshaft periplate	3	6	Distal femoral plate (DFP) over DFP
2	56	Female	Right	Proximal humerus	06/7/2010	Midshaft periplate	7	7	LCP over PHILOS plate
3	85	Female	Right	Distal femur	22/5/2012	NOF extra-capsular	7	7	Proximal femoral plate over DFP
